# Resilience, tipping, and hydra effects in public health: emergent collective behavior in two agent-based models

**DOI:** 10.1186/s12889-016-2938-8

**Published:** 2016-03-15

**Authors:** Christopher Robert Keane

**Affiliations:** Behavioral and Community Health Sciences, Graduate School of Public Health, University of Pittsburgh, 130 DeSoto Street, Pittsburgh, PA 15261 USA

## Abstract

**Background:**

Collective health behavior often demonstrates counter-intuitive dynamics, sometimes resisting interventions designed to produce change, or even producing effects that are in the opposite direction than intended by the intervention, e.g. lowering infectivity resulting in increased infections. At other times collective health behavior exhibits sudden large-scale change in response to small interventions or change in the environment, a phenomenon often called “tipping.” I hypothesize that these seemingly very different phenomena can all be explained by the same dynamic, a type of collective resilience.

**Methods:**

I compared two simple agent-based models of interactions in networks: a public health behavior game, in which individuals decide whether or not to adopt protective behavior, and a microbial-level game, in which three different strains of bacteria attack each other. I examined the type of networks and other conditions that support a dynamic balance, and determined what changes of conditions will tip the balance.

**Results:**

Both models show lasting dynamic equilibrium and resilience, resulting from negative feedback that supports oscillating coexistence of diversity under a range of conditions. In the public health game, health protection is followed by free-riding defectors, followed by a rise in infection, in long-lasting cycles. In the microbial game, each of three strains takes turns dominating. In both games, the dynamic balance is tipped by lowering the level of local clustering, changing the level of benefit, or lowering infectivity or attack rate. Lowering infectivity has the surprising effect of increasing the numbers of infected individuals. We see parallel results in the microbial game of three bacterial strains, where lowering one strain’s attack rate (analogous to lowering infectivity) increases the numbers of the restrained attacker, a phenomenon captured by the phrase, “the enemy of my enemy is my friend.”

**Conclusions:**

Collective behavior often shows a dynamic balance, resulting from negative feedback, supporting diversity and resisting change. Above certain threshold conditions, the dynamic balance is tipped towards uniformity of behavior. Under a certain range of conditions we see “hydra effects” in which interventions to lower attack rate or infectivity are self-defeating. Simple models of collective behavior can explain these seemingly disparate dynamics.

**Electronic supplementary material:**

The online version of this article (doi:10.1186/s12889-016-2938-8) contains supplementary material, which is available to authorized users.

## Background

Collective health behavior often demonstrates counter-intuitive dynamics. Sometimes, collective behavior does not respond to interventions designed to produce change. At other times, collective health behavior may show extreme responsiveness to a new small intervention or, more generally, exhibits sudden large-scale change in response to some small change in the environment, a phenomenon often called reaching a “tipping point” [1–3] or “tipping the balance.” In a population, this tipping can result in a rapid spread of a behavior that defies standard predictions. For example the change may not occur when expected, or the direction of change may be in the direction opposite to that expected. In fact, interventions in populations sometimes have the opposite effect than predicted, a phenomenon known as “policy resistance,” the “tendency for interventions to be defeated by the system’s response to the intervention” [4–6]. This counter-intuitive response of a system is also known as the “hydra effect,” named after a mythological multi-headed serpent that grew two heads for every one that an individual cut off [7–9]. For example lowering infectivity can result in increased total infections. Interventions that lower risk can increase adverse outcomes due to “risk compensation,” e.g. lower perceived risk due to antiretroviral therapy (ART) can increase sexual risk taking; [10] lower perceived risk may lead to a premature drop in health protective measures; [11] antibiotics resulting in increased infections due to increased resistance [12], harm reduction can actually increase negative consequences [13–15] In these cases, an intervention to reduce infectivity (ART, antibiotic, harm reduction) can have the opposite than expected effect.

These seemingly very different dynamics, I propose, can all result from a single type of ecological resilience. Public health behavior is part of a social ecological system, because it involves interaction between individuals, and this interaction affects mutually shared conditions. Moreover, as we will see, health behavior can be subject to negative feedback, also called “balancing feedback” that tends to regulate behaviors at the population level. Under these conditions of shared conditions and balancing feedback, counter-intuitive dynamics often result, such as stability when we would expect change, or change when we would expect stability.

I will attempt to show that these various counter-intuitive dynamics may stem from a model in which health conditions are shared, and in which there is negative feedback in the form of health behavior that responds to disease. My aim is to provide a model that provides one general explanation for a variety of effects such as resilience, tipping and hydra effects. I present and compare two related models of collective adaptation and dynamic balance that can explain certain forms of policy resistance resulting from a robust dynamic equilibrium. I propose that these models also show how “tipping” results when some initial state of dynamic balance is disrupted. Specifically, dynamic balance may seem relatively invisible until some change, e.g. a minor change in a network or in perceived benefit of a behavior, upsets a dynamic balance. The models show how, through collective adaptation, a population can retain a diversity of behaviors that remain resilient against some kinds of changes, whereas other kinds of changes will tip the balance such that one behavior takes over.

For the above purposes, I offer a new repeated “public health game” in which individuals choose to protect health or not. In this model, benefits of health are shared locally in a network. Health is a shared benefit when an individual, by virtue of being healthy, confers benefit to local contacts who transact with that person. Every individual benefits from having healthy neighbors because having healthy contacts allows the individual to engage in beneficial transactions for work, recreation, financial, physical and emotional support. There is abundant evidence that having healthy alters (others) in one’s network benefits ego. Higher amounts of social support is associated with increased health in general [16–18]. Thus, we assume in our model that one’s healthy neighbors are beneficial. More formally, an individual ego that adopts health protective behavior gains health and also benefits the ego’s nearest neighbors (alters). Each individual ego earns benefit for each healthy person in their local neighborhood (ego plus alters make up a local neighborhood). Each individual can either protect their health or not protect (defect). A “protector” essentially puts up a shield to prevent spread of infection. Protectors pay a cost in time, effort, or some other negative consequence of taking protective action, but will be healthy as result of taking the action. That is, protection assures health as long as one protects but incurs some immediate perceived cost, including time required to perform the behavior, e.g. time to wash hands, discomfort or embarrassment in wearing a facemask, or using a condom. “Defectors” pay no cost, because they are not taking protective action, but they will become infected if they have any infected neighbors.

This model I present applies to behaviors that protect against spread of illness while that behavior is adopted, e.g. facemasks prevent spread of respiratory illness while used (by contrast vaccination lasts well past the time of behavior). The public health model I present in this paper thus applies to non-pharmaceutical protective behaviors such as facemask use, handwashing, or condom use, all known to reduce transmission of infections [19], and applies to illnesses for which there is not lasting immunity. Several transmissible diseases, often caused by bacteria, do not produce an immune response in the body, e.g., sexually transmitted such as gonorrhea, syphilis and chlamydia. For these bacterial diseases, after antibiotic treatment is completed, individuals return to an unprotected, susceptible state. Humans also are unlikely to develop full immunity to some viruses, such as rotavirus and rhinoviruses that cause acute respiratory infections and the common cold [20]. There are more than 100 recognized serotypes of Rhinovirus, the primary cause of common cold, and so individuals are unlikely to develop full immunity [20]. Individuals can contract rhinovirus up to five to eight times a year [21]. Recurrent protective behavior is necessary to prevent recurrent illnesses that spread.

Likewise the costs of protection associated with behaviors such as handwashing or facemask wearing are proportional the period of the behavior, and recur with repeat behaviors. Individuals perceive costs of using facemasks to include discomfort, ill-fit, inconvenience (when wanting to eat, speak or show facial expression etc.), or embarrassment [19–24]. Perceived costs associated with handwashing include the time it takes, distance to water or skin irritation [25–30]. Perceived costs associated with condoms include discomfort, violation of perceived ethnic and religious norms against condoms, perceived stigma of condom use, and anxiety discussing condoms due to lack of trusting relationship with a partner [31–37]. These costs and benefits recur when the protective behavior recurs, and cease when the protective behavior ceases (true for handwashing, facemask wearing and condom use). Several theories of health behavior change, such as the health belief model and transtheoretical (stages of change) model, posit that the costs and benefits of health behaviors influence health behavior, and these theories have been successfully applied to handwashing, facemask wearing and condom use [30, 38–41]. Likewise, individuals in the agent-based public health model I present consider costs and benefits of behaviors, but individuals learn the sum of costs and benefits of behaviors by observing neighbors’ current action and payoff. In my model, individuals only consider the current situation, the current round of play. Healthy defectors surrounded by only healthy individuals experience the benefit of health but do not consider the risk of infection, because they see no neighbors currently infected. This roughly corresponds to the “pre-contemplation” stage of the stages of change model [38–43], in which individuals do not consider the costs of the unhealthy behavior. But defectors are likely to become infected, at which point they experience the cost of defection (loss of health), roughly corresponding to the “contemplation” stage. Infected defectors next to one or more health protectors may see that protection at this point earns a higher payoff than defection. This corresponds to the “determination” (or preparation) stage in which an individual determines a behavior to adopt that leads to a better outcome. Individuals who then adopt protection are in the “action” stage. If a health protector is surrounded by other health protectors, they gain positive social support (earning a benefit roughly proportional to the number of healthy neighbors). So long as a protector does not see defectors with higher payoffs, the protector is not tempted to defect, and protection is maintained, corresponding to the “maintenance” phase. But a protector defects if they are next to a defector earning the highest payoff, corresponding to “relapse”. These examples, show how individuals might move through stages of decision making based on costs and benefits learned from neighbors in a network.

Each person can only interact with their neighbors in a network (neighbors are those one step away, local neighbors or “nearest neighbors”) [44]. Every individual earns a total value (payoff) that is the total benefit minus the cost. Everyone then adopts the behavior of the local neighborhood person with the highest current payoff. In the real world, individuals may differ in what they subjectively perceive as the highest payoff, but in this simplified model the perceived success is equivalent to the payoff we defined above which may represent perceived health and happiness of individuals. Yet imitating the person with the highest payoff is inherently subjective, and is usually classified as a “bias.” Adopting the behavior with the locally highest payoff, also called highest payoff bias, “imitate the best,” [45] or copy-successful-individuals [46, 47], is common among humans and animals [47–53] and is relatively adaptive to changing local conditions despite that it biased [45, 46, 54, 55]. It likely is very costly or not possible to monitor much more than this local current sample and that it is likewise costly to try to learn health behavior through trial and error (perhaps getting sick along the way) [46, 56, 57], therefore we have our simulated individuals adopt the behavior with the locally highest payoff. If no local person has a higher payoff than oneself, one does not change behavior. All these steps in the game are repeated, allowing the spread of behavior over time in the network.

Repeating this game model may reveal surprising results in the longer term. In the short run, it would seem that clusters of healthy defectors benefit the most because they pay no cost and also have many healthy neighbors. An individual can receive a benefit of healthy neighbors without paying a cost, which is “defecting” or free riding on protectors. However, the very success of defection can be its undoing, due to spread of infection to defectors. This is a negative feedback effect that leads to a version of the classic tragedy of the commons [58]. Defection spreads widely at first, but then tends to slow down or collapse entirely. The spread of defection triggers a self-limiting negative feedback because defection lowers its own source of benefit (healthy neighbors). The ensuing rise in infection should, in turn, lead to more protection. This rise and fall of infection, defection and protection could produce a dynamic balance resulting from the negative feedback of protection responding to infection. This dynamic balance may result in resistance to outside interventions because a rise in protection may be countered by a rise in defection and thus a rise in infection. In fact, since defection can sometimes be the victim of its own success, an intervention that would seem to help defection and the spread of infection, may actually increase infections in the long run. To see whether or not this is the case, we will run the public health game repeatedly while varying the conditions such as benefits, costs, infectivity, and network structure. It will also be instructive to compare the results of the public health game to a very simple case of dynamic balance that we can illustrate with a simple repeated game. This game reveals how even seemingly lethally competitive individuals, three strains of E. coli bacteria, can coexist, in a non-linear dynamic equilibrium [59, 60]. Thus both models, the public health game and the microbial rock-paper-scissors game, may show a similar resilience resulting from negative feedback. Prior empirical research has demonstrated that the rock-paper-scissors dynamics produce a kind of resilience, resulting from a type of negative feedback process which, in turn, results in counter-intuitive responses to lowered infectivity [61]. Such a counter-intuitive effect may also be present in the public health game.

## Methods

I simulated the above public health game in several types of networks. Each simulation run begins by randomly filling the network with 50 protectors and 50 % defectors. In addition a random 10 % of individuals have a contagious infection. Thus, the game starts with a random distribution with 50 of individuals protecting, 50 defecting, and a random 10 % infected. Each healthy individual gets a unit benefit *b* for being healthy and confers a benefit to each local contact who is healthy As a result, everyone gets a unit benefit *b* for each local person (self and neighbors) who is healthy.

Why does one’s health benefit one’s neighbors? The public health game assumes that physical health benefits not only the healthy individual but also neighbors, by allowing instrumental social support of those neighbors, helping to perform functional activities or joining in recreational activities. Conversely, a temporarily debilitating disease, such as influenza or even a severe cold, limits the instrumental support the ill person can offer to contacts. Moreover viral infections are associated with depression [62, 63], thus impairing emotional support an infected person can offer. In contrast, positive mood helps support others emotionally, for example a study found each happy contact increases the probability per year of becoming happy by 11 % [64]. Moreover, healthy mood spreads through networks and healthy mood among friends is associated with significantly reduced risk of developing and increased chance of recovering from depression [65]. Thus the benefits of health are shared with one’s contacts, partly as instrumental and emotional social support.

Protectors must pay a cost *c* for each neighbor. This is an abstraction of the idea that in non-pharmaceutical measures such as handwashing and facemask wearing, the behaviors are roughly proportional to the number of one’s contacts. Once one is away from all contacts, one need not wear a facemask or wash hands. If an individual encounters contacts only during a brief visit to a grocery store, one puts on a mask during that time, and washes hands afterwards. However, if one comes in contact with many others throughout most of the day, one must wash hands or wear a mask throughout the day. Somewhat similarly, one who has multiple sexual contacts must more repeatedly use a condom.

A person who chooses to protect will not contract an infection from a neighbor. If an infection is present in an individual, it will spread to any neighbor who is not protecting (defecting). We set the duration of infection to 5. This is a simplified susceptible-infected-susceptible (SIS) model in which individuals are susceptible again after an infection that lasts for 5 steps (game plays). Because there is a spreading disease, one can be healthy either because they protect against the disease or because the infection is not present locally.

At every round each individual adopts the local behavior with the highest payoff, then recalculates their payoff. Individuals change behavior only if one of the locals with a different behavior than one’s self is earning more than any other local (including one’s self). We repeat this sequence. Running the game in with many repeated plays, and with many variations of conditions, requires a computer simulation. For this purpose I programmed the game simulation in NetLogo [66, 67]. I simulated this game in networks of size 61 by 61 (3721 individuals).

I repeated the public health game for each of a range of ratios of *b* to *c*, always keeping *c* = 1, then increasing *b* from 1 by .01 increments. I started with *b* = 1 then run the simulation for 500 steps (game plays). Then I increased *b* to 1.01, then simulated the game for 500 steps, and so on for higher levels of *b*. I also varied the levels of infectivity, holding the level of benefit and cost constant. I set *b*/*c* to equal 5. Each person gains *b* = 5 for every local person who is healthy, holding *c* equal to 1. Then I varied infectivity from 100 % (infection will always spread from an infected person to neighboring defectors) down to 0 % (infection does not spread), incrementing the likelihood of infection transmission by 1 % at a time. For each of the resulting 101 levels of infectivity, I simulated the game for 500 steps (game plays), calculated the mean proportion infected, and plotted the results. Having one distinct value for each level of benefit, and each level of infectivity is a simple way to revealing the bimodal results (as we will see in the resulting plots of infectivity by proportion infected). However it is also useful to have averages for each level of infectivity. Therefore, for each of the 101 values of infectivity (0–100 %), I ran 10 simulations of 500 steps each and averaged the proportion of infected persons. This produced 101 mean values of proportion infected over 500 steps.

### Clustered networks versus random networks

I compared the effects of type of population contact structure by running simulations for different types of networks, a random network and a square grid lattice with varying levels of clustering. In each network type, every individual has eight neighbors. The square grid network naturally incorporates typical spatial clustering of individuals and their conditions. In the square grid lattice network, individuals can interact with their eight nearest neighbors, much as checkers can interact with their immediate neighbors on a board (north, south, east, west, plus the four corners at the diagonals), and the larger checkerboard grid structure results in a clustering coefficient of 42.9 %. This means that for any individual in the grid, the proportion of one’s neighbors that know each other is 42.9 %. This degree of clustering of the networks we examine approximates that found in many real social networks [68, 69] and has often been found to be the underlying network structure in which various health behaviors spread [70, 71]. I also ran simulations for grid lattices with lowered levels of clustering. To lower the clustering coefficient, I used an algorithm [68] that randomly rewires a number x of the connections in the square grid lattice, while preserving an average *K* = 8. The rewiring process deletes a link in the square lattice, and replaces it with a randomly selected link. The algorithm iteratively finds reconnections until x number of connections are rewired. However, the algorithm does not change the total number of neighbors each individual has. The outcome of this process is to lower the clustering coefficient, the degree to which each individual’s neighbors know each other, conferring small-world properties to the network. Our analysis focuses on these networks with small-world properties. Many social networks are structured as small-world networks, in which the average path length between pairs of individuals is relatively low, and yet the clustering remains relatively high, close to the level in a grid lattice [68, 69].

In the random network, individuals are randomly connected to each other, but to compare to the grid lattice network we make sure that everyone only has an average of eight neighbors as in the grid lattice. However, in the random network, these links are generated by randomly connecting everyone to eight other persons in the network, on average. Thus, the random network is the same as the grid-lattice in terms of average numbers of neighbors, but in the random network one’s neighbors are extremely unlikely to know each other (very low clustering). In other words, the random network, the average proportion each individual’s neighbors who know one’s other neighbors is nearly zero, whereas in the grid-lattice network there is a high degree of neighbors knowing each other (very high clustering). Health behaviors spread differently in a random versus a grid-lattice network. For example, an experiment manipulating network structure found that health behavior spread further and faster through clustered-lattice networks compared to random networks [70]. Thus, our comparison of a random vs. a grid-lattice network is likely to show different dynamics in the spread of behavior.

### Microbial rock-paper-scissors simulation

The microbial simulation involves three strains of e-coli on a surface containing nutrients. One strain produces antibiotic which kills one other strain. A third strain is resistant (“resister”). The strain that is vulnerable to the antibiotic “producer” has its own strength, namely it can grow rapidly (“grower”). Each strain can outcompete one other strain, and in that sense are like a rock-paper-scissors situation: Producer beats grower; Resister beats producer; Grower beats resister. The winning strain reproduces in the space occupied by the losing strain, so as to consume the nutrients of the losing strain. But what happens when all three strains are together in a petri dish or on a stretch of tissue? Imagine each individual microbial cell occupies one patch of a grid lattice network. One can try this on a checkerboard by covering each patch on the checkerboard with a chip of one of three colors (red, green or purple). The chips of each color are in equal numbers, each making up one-third of the total, but they are distributed across the patches in the grid at random. Each chip represents one of three types of an e-coli bacterial cell surrounded by eight other cells. Have each individual bacteria play the game with all their eight local neighbors. Each individual receives 1 unit benefit *b* for every neighbor it beats. For example, if the individual player is a Rock surrounded by eight Scissors then the Rock wins eight points total in payoff. After everybody gets their payoff, each individual adopts the behavior of the highest scoring individual in the neighborhood, including self and their eight neighbors. This picking out of the locally highest payoff is the same general strategy we used in the public health game. We can think of this as the bacterial strain spreading, or a behavior spreading. In the case of the e-coli, the winning local strain reproduces in the local spaces of the losing strain.

In order to run this game for a variety of conditions, I programmed the game in NetLogo software [66]. I ran the rock-paper-scissors game in the same sized networks as for the public health game (3721 individuals). As for the public health game, I compared a random network to a grid lattice network (61 by 61 size grid), and also varied the clustering levels in the grid by gradually adding random structure to the grid network, rewiring local connections as described earlier. For each value of non-local interaction, ranging from 0 to 5 %, I plotted the final points after T = 500 rounds of play. That is, I ran one simulation for 600 rounds of play with 0 % non-local interactions. Then I ran another simulation for T = 500 points with the proportion non-local fixed at 0.1, and so on through 5 % non-local interaction.

To determine the effect of decreasing the producer strain’s attack rate, I ran the model 500 times for each of levels of attack rate, ranging from 0 % (no attack, thus no point gain when encountering grower) to 100 % (always attack when encountering grower, and always gain a point for each encounter). I program recorded the mean levels of each strain for each run of 500 steps.

## Results and discussion

### Thresholds and oscillations in the public health game

**Fig. 1:**
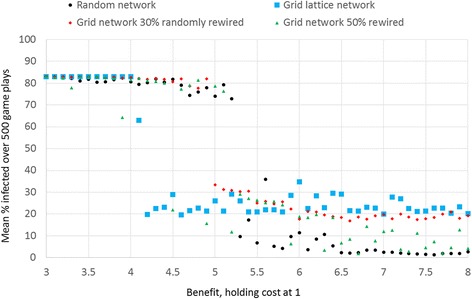
Proportion infected by benefit of health and network type. For each level of benefit b shown, I ran one simulation run lasting 500 steps (game plays) and calculated the averaged the proportion infected. The simulation runs all start with a random distribution of 50 protectors and 50 % defectors, and 10 % of individuals infected. In the grid lattice network, when the health benefit is below 4, health protection and so infection spreads unabated

In the grid lattice network, protection cannot survive when the benefit to cost ratio is below 4, and so infection takes over (Fig. 1). Benefit to cost ratios larger than 4 in the grid network allows protection to survive, but the levels of protection, defection and infection oscillate, with the average level of infection at about 25 %. That is, the game produces oscillations of infection, protection and defection, so long as the benefit is 4 or higher, when cost is equal to 1. Decreasing the b/c to less than 4 is what tips the balance towards the collapse of protection, and the takeover of infection.

In a completely random network, the level of benefit to cost ratio must be at least 5 for protection to survive. Thus, compared to the grid lattice, the benefit must be higher in the random network to support protection. However, in the random network, protection often completely takes over, wiping out infection completely, when the b/c is above 5 (Fig. 1). That is, in the random network, when b/c is above five, protection either completely takes over and wipes out infection, or protection dies, leaving infection at the highest levels.

In the intermediate networks with 30 % of connections randomly rewired in a grid lattice, the b/c level must be five or more for protection to survive, oscillating with defection and infection, much like the grid lattice, but the average levels of protection are slightly lower (Fig. 1). Randomly rewiring 50 % of connections results in a more random relationship with benefit, but the proportion infected is relatively low for higher levels of benefit. That is because a more randomly wired network is less likely to support oscillations of protection, defection and infection. In sum, the completely random network produces no oscillations, whereas the grid lattice network does support these oscillations. Let us examine the nature of these oscillations.

**Fig. 2:**
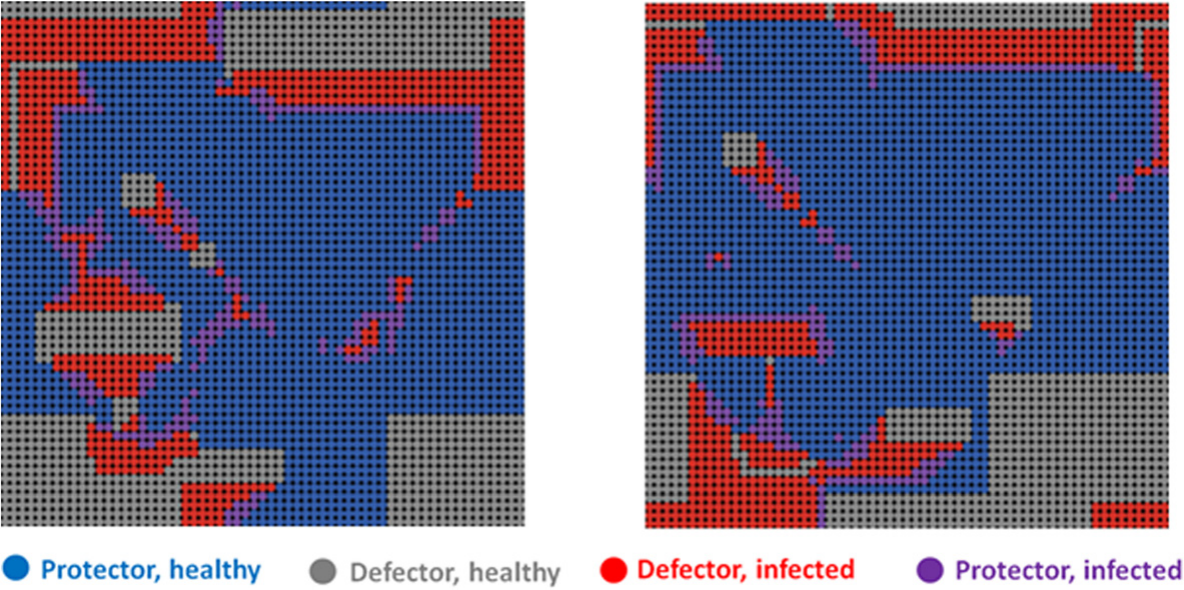
In a grid network, we start with a random distribution of 50 protectors and 50 % defectors, and 10 % of individuals infected, infectivity = 100 %, *b* = 5, *c* = 1. The left panel shows an example of the spatial configuration of protection, defection and infection. This is the pattern at time = 320 steps. The right panel shows the situation at five steps later, T = 325

In the grid lattice network, when the benefit to cost ratio is above 4, infection spreads among the defectors in the early steps of the game, but by about 100 steps, a substantial group of individuals learns to protect. A smaller proportion learns to free-ride on the protectors, defecting. Infection thrives on these defectors so a surge of infection follows a rise in defection, and this is then followed by protection. After an initial period of learning, the peaks consistently follow a pattern of protection, defection, infection. To visualize why this cycle occurs, examine the patterns of local spatial change in the square grid (Fig. 2, compare left and right panels). We see that when disease is not present locally, defection rises. That is because some individuals who defect not only can free-ride on healthy persons, but also are relatively likely to be healthy themselves even while not paying the cost of protecting. It is an advantage in the short run to defect in this situation so that the number of healthy defectors rise, with these defectors bordering the protectors. But soon this results in disease rising and we see a sharp rise in infected defectors. That is because the advantage of defecting is only temporary. At first defectors are surrounded by enough healthy persons to have a relatively high level of benefit, but often what happens is defection spreads then disease takes over. As disease spreads, protection again becomes an advantageous behavior. Thus protection rises. Put another way, there are three kinds of negative feedback operating: (1) infection lowers the level of healthy defectors when healthy defectors rise high; (3) protection lowers infection after infection rises high; (3) defectors rise in response to the surge in protectors.

**Fig. 3:**
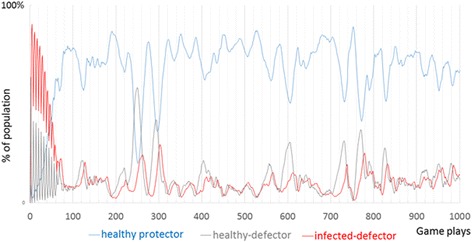
The public health game over 1000 steps. After an initial period of adaptation, three types consistently follow one after another: protection, then healthy defection, then infection

As a result we see a cycle: a rise in protection, then a rise in defection then a subsequent rise of infection. Specifically, when disease drops, protection soon drops in response. We then see a rise of healthy defectors, followed by infected defectors (and a very few infected-protectors who may still have not recovered from the disease they contracted before they decided to protect). We see this pattern spatially (Fig. 2) and temporally (Fig. 3). Defectors always move in where there were protectors, these defectors are generally healthy, and can free ride upon the protection of the protectors. Then infection spreads to the defectors (Fig. 2). A few defectors decide to protect even before they have recovered from the disease. The overall result is a spatial movement in the network in which the groups consistently follow one another in the grid network: protection, then healthy defection, then infection. This type of spread from neighborhoods to neighborhoods is possible in a network with high local clustering, where there is thus much shared feedback about the local situation. This cycle, often lasting very long, is evident in the overall population trends shown in Fig. 3.

### Clustering, collective feedback, change at the margins

The extent to which a behavior can spread through a network is a function not only of the behavioral rules of the game, but also a function of the type of network. The benefit of having healthy neighbors depends not only upon the number of healthy neighbors but also depends upon whether or not one’s healthy neighbors are connected to each other. When many neighbors are connected to each other, they are more likely to share health conditions and information about those conditions. When one’s neighbors protect health, those neighbors are also helping each other. This leads to a kind of spatial reciprocity of the same type that occurs in a variety of cooperative games [72, 73]. Feedback to any given individual comes from that individual’s local neighbors, a local collective feedback, meaning that individuals can see the behaviors and the associated payoffs of local individuals.

In the grid type of network, it is possible for behaviors to cluster together. Imagine there are only 25 protectors in the entire grid network. These protectors can form a 5 by 5 square block of protectors, such that the person in the very center has eight protecting neighbors that are themselves surrounded by 16 protectors. However, in a random network with 25 persons protecting, it is unlikely that a protector has protecting neighbors who are in turn themselves surrounded by only protectors. This is one way that protection can form as a collective action in a grid network, but not in a random network. While any individual benefits from taking protective action, the shared benefits can be more concentrated in a clustered network which makes it easier to spread. Where a cluster of protectors meets a cluster of defectors, defectors at the margins switch to protection (Fig. 2, comparing the left panel to the right panel). As a result, the collective of protectors expands. The positive example of this cooperative protective action can spread in a clustered network. Unfortunately, defection can thrive where there are many protectors. For this reason, a cluster of protectors can be replaced gradually with a cluster of defectors. With enough defectors infection follows, creating a cycle in a grid network, resulting from collective negative feedback.

### Paradoxical effects of increasing infectivity

**Fig. 4:**
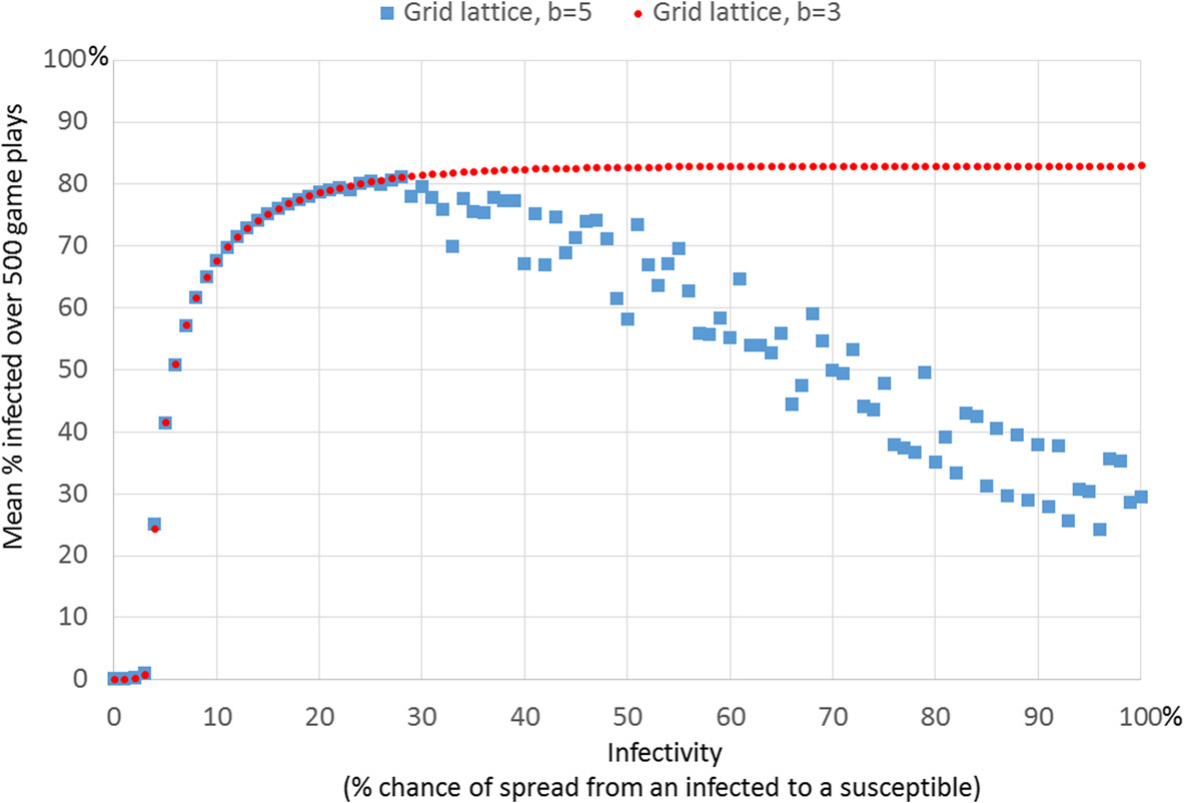
Hill-shaped relationship between average proportion infected and infectivity when benefit is above the minimum threshold allowing protection to survive. This plot shows the average of 10 simulation runs for every level of infectivity. Each simulation lasted for 500 steps

The situation described in the prior section also produces results that at first seem paradoxical. A decrease in infection eventually produces a cycle of events that lead to increase in infection, in a clustered network. Likewise, were we to intervene by reducing infectivity of the microbe, we can reduce individual’s perceived need for protection. Running 500 runs of simulations repeatedly for each varying infectivity from zero to 100 %, when *b* = 5, shows these surprising results (Fig. 4). Increasing infectivity from zero to about 29 % results in a higher average level of infection, but raising infectivity higher and the infectivity begins to lower because protection kicks in to lower infection. The average benefit from health experienced by an individual is a function of the number of local persons who are healthy, which we can lower by either lowering benefit or by lowering the chance of infection (infectivity). When holding benefit constant, therefore, below a certain threshold level of infectivity, about 30 %, the benefit of protection does not outweigh the costs. Above this threshold, a kind of tipping point, higher infectivity means that protection becomes more likely and this reduces infection. This result is another case of negative feedback kicking in, once a certain level of infection occurs, protection is likely to reduce the infection.

**Fig. 5:**
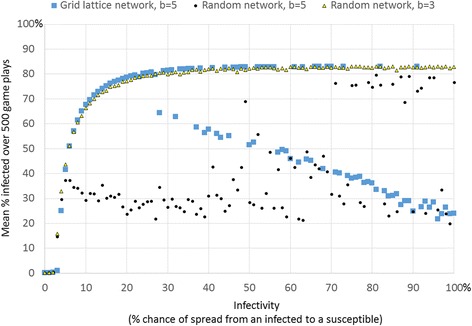
There is a bimodal level of average infection in the grid network when benefit is above the threshold allowing protection to survive. For each level of infectivity, this plot shows one simulation run (whereas Fig. 4 averaged results over 10 simulation runs), which makes the bimodal average infected evident when *b* = 5 in the grid network. The plots also show average proportion infected by infectivity for other network types. In a grid lattice network, when infectivity is 29 % or higher, health protection was increasingly likely, and thus infection was increasingly lower. There are two distinct average levels of infection, one for which protection survives, and another for which protection dies out early. In a random network, rapid spread of disease early in simulation runs triggers protection which in turn lowers infection

When the benefit *b* is lower than the threshold necessary for protection to take hold (*b* = 4 for a grid network or *b* of about 5 for a random network), then the mean proportion infected rises with increasing infectivity, as would be expected (Fig. 4, for *b* = 3). However, when the benefit is above the threshold needed for infectivity to take hold (Fig. 4, for *b* = 5) then the slope of the relationship between infectivity and proportion infected is hill shaped (Fig. 4, which averages 10 simulation runs per level of infectivity). The slope is positive for lower levels of infectivity (below about 29) and then negative for higher levels of infectivity (infectivity above about 29 %). Moreover, the mean levels of proportion infected are bimodal. Above values of 30 % infectivity, each simulation run produced one of two distinct values of mean proportion infected, either about 80 % or a much lower level that decreases linearly with level of infectivity (Fig. 5). The bimodal results are easily seen when we plot one mean level of infection per level of infectivity (as in Fig. 5). The higher level of mean infected (about 80 %) results when protection collapses early and so the infection takes over. The lower level occurs when protection survives. When protection survives, it does so in linear proportion to the degree of infectivity, with higher protection for higher infectivity. This occurs because the payoff for protection versus the payoff for defection increases with the chance of infection. This is true when *b* is sufficiently high. Figures 4 and 5 shows the resulting curves. When the benefit is set low, e.g. *b* = 3, then protection never takes hold so the proportion of infected is simply a function of infectivity (Figs. 4 and 5).

### Rock-paper-scissors game in microbial agent interaction

**Fig. 6:**
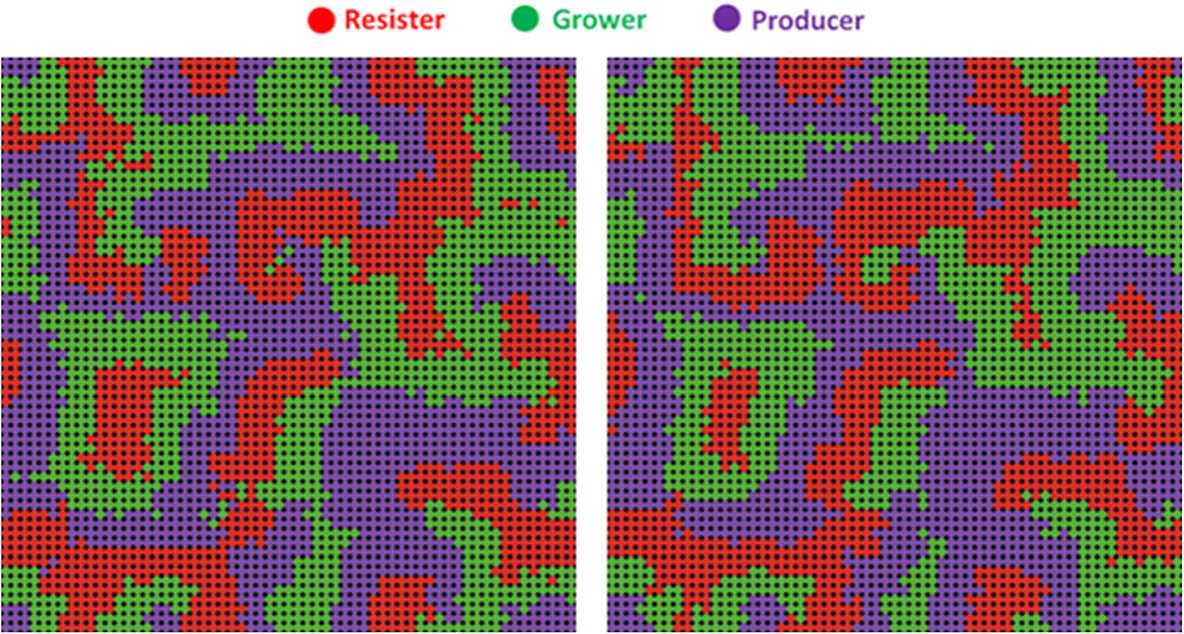
Left: Bacterial rock-paper-scissors at step 100 (left) and at step 101 (right)

**Fig. 7:**
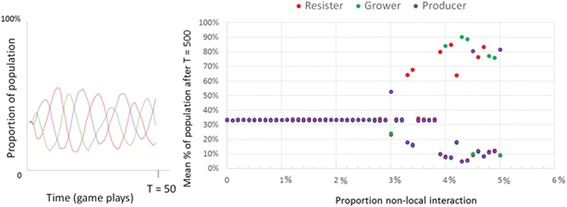
Left: Cyclic dominance over the first 50 steps (in a grid with no rewirings). Right: Long-term results for rock-paper-scissors game plotted against the proportion of local connections rewired. These are the average proportions after 500 steps

The public health game produced a pattern of cyclic oscillations (Fig. 3) that roughly follows what is known as a rock-paper-scissors cycle, a cycle in which the frequency of rock peaks, then the frequency of paper peaks (because paper beats rock), then the frequency of scissors peaks (because scissors beats paper) and this cycle repeats. At the local level we see collective clusters of rocks gradually take over a collective cluster of scissors and so on (Fig. 6), consistent with empirical findings of the e-coli on a surface [59, 60]. At the global level, each of the 3 strains will take a turn having a higher proportion than the rest, but none will overtake the others completely. Rather, each will average 33.3 % of the total in the long run (Fig. 7). Each strain, each with its own behavior, exists in local collective clusters, which battle each other. However, at the global and long-term level we see a balance, with each strain taking up an average of one third of the total population.

An important part of the explanation for the resilience and cyclic dominance in the spatial rock-paper-scissors game is the protective function of collectives. Collectives in a grid can form a spatial shielding of community members from exposure to outsiders. If we change that grid structure by “rewiring” local connections, replacing those with random connections, we can disrupt that spatial protection and disrupt the cyclic dominance (Fig. 7, right). We gradually rewire more and more connections to determine the point that the cyclic dominance is disrupted. After a certain number of rewirings, between 3 and 4 %, the dynamic equilibrium tips over. Above about 3–4 % of disruption of local clustering (Fig. 7), we see the system tip to complete dominance of one of the strains.

### Paradoxical effects of lowering the attack rate

**Fig. 8:**
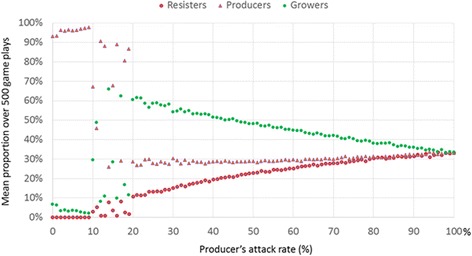
Lowering producer’s rate of attacking grower increases the producer’s abundance, and decreases the abundance of the grower strain

Another way to tip the balance is to lower the attack rate of any strain. We will focus on the attack rate of the producer. Recall that the producer wins over the grower (because the grower is sensitive to the antibiotic made by the producer). If there were only a pairwise competition between producer and grower, then lowering the producer’s attack rate would only slow down the producer’s rate of overtaking the grower. However, when the three strains are operating, the effects are the opposite. Dropping down the producers attack rate below 20 % dramatically increases the numbers of producers (Fig. 8). When the producer restrains its attack to this very low level, then the susceptible strain grows very quickly and wipes out the resistant strain. With the resistant strain gone, the producer’s growth is unrestrained (Additional file 1: Figure S2). This is because the producer’s enemy is the resistant strain. The enemy of the resistant strain is the susceptible strain (“grower”) which is normally attacked by the producer of the antibiotic.

### Increasing cost of antibiotic production

So far we have assumed that the costs of each strategy in the rock-paper-scissors are all negligible (and so all equal). There is a cost to producing the antibiotic, a cost to growing more rapidly, a cost to resisting antibiotic. When these costs are equal, then each of the three strains averages at 33 %, following the usual rock-paper-scissors dynamic. We can first set the costs of all three strains to equal one, then vary the relative costs, e.g. lower the costs of one of the strains, to see the effects. What would happen if we lower the costs to producing antibiotic? One naively may assume that this would increase the success of the antibiotic producer. It turns out that reducing the cost of the producer increases the proportion of resisters to almost 40 %, on average, and decreases the proportion of growers to about 28 %, whereas the proportion of producers remains at about 33 %. This is true for cost reductions of 10 % down to 90 %. Reducing the cost by 100 % further increases the proportion of resisters to about 45 and decreases growers to about 22 %. If there were only two strains, say producer and resister, this counter-intuitive result would not hold. But when there are three strategies, then giving a cost advantage to any of the strategies in this rock-paper-scissors game, has the effect of increasing the numbers of one’s enemy, due to there being three negative feedback dynamics, which together form a new kind of negative feedback. So increasing the cost advantage of the antibiotic producer actually increases the number of resisters (Additional file 1: Figure S1).

## Conclusions

The “public health game” demonstrated that when benefits of health protection are shared locally in a network, counter-intuitive dynamics result. First, this game produces a lasting dynamic equilibrium: health protection, disease, and free-riding take turns predominating. The public health game shows that, under a wide variety of conditions, negative feedback in local neighborhoods tends to result in toleration of coexistence of both protectors and defectors. Negative feedback in networks with local clustering leads to nonlinear trends and often coexistence of cooperative collective health protection and free-riding in dynamic equilibrium. Likewise, in the bacterial rock-paper-scissors, despite the fact that each strain kills another strain, all three strains co-exist in cyclic dominance of tolerance and constant adaptation. A variety of behaviors among humans and animals also follow this rock-papers-scissors patterns or similar forms of a cyclic dominance, which can involve three or even more than three behaviors [74–76]. A relatively simple case of this population rock-paper-scissors dynamic occurs when microbes compete for resources, inspiring the microbial model simulations we examined, which corresponds to a dynamic seen in real e-coli, when the three strains interact on a surface of nutrients. Pairwise in vitro competitions between each of the three e-coli strains shows each of the three rock-paper-scissors rules to be empirically true [60]. The researchers took only two strains at a time in laboratory experiments, and confirmed these simple rules. If we only put resister and producer together, resister is happy to reduce producer down to zero. Now the question is what happens when all three strains are together. One might expect that the antibiotic producer would kill the fast growing but sensitive bacteria, but it turns out that the three strains can coexist in dynamic equilibrium in our model and in the empirical petri dish tests [59, 61], a pattern in which each strain predominates for a brief while, but none completely overtakes the test. The three negative feedback loops together form a new kind of negative that balances all three over time. The result is cyclic dominance, with each strain taking turn dominating but none taking over completely [59, 61]. This is a type of resilience, meaning the system returns to a balance after a deviation [77].

Crucially, the cyclic dominance we saw in the rock-paper-scissors game depends upon local collective interaction, upon bacteria interacting only with local neighbors, and then those neighbors interacting with their local neighbors. Bacteria on a surface of a petri-dish or a gut have local neighbors in a network, somewhat like the grid network we have examined. Researchers have tried shaking the flask of bacteria to break up the local effects, similar to randomly rewiring local links. That disrupts the balance, prevents the establishment of biodiversity; eventually one strain takes over [59, 78].

Both games show adaptive local community resilience, but also show “policy resistance” in which interventions can be self-defeating. A more colorful metaphor for this effect, often used in ecology [8] and sometimes in political science [9], is the “hydra effect,” where cutting of any of the several heads of the monstrous hydra results in more heads appearing than even before. Such a variety of effects, growth and change when we expect stability, and stability when we expect change, can result from a single form of balance resulting from negative feedback in both of the games. This balance, resulting from negative feedback (also known as balancing feedback), can be tipped by disrupting local community structure or by changing the level of infectivity, analogous to an attack rate. In both cases the results of the tipping can be counter-intuitive. Due to negative feedback, lowering infectivity below a certain threshold (tipping point) can increase the total infection. The most dramatic effect is seen when dropping infectivity from a number close to 100 % to about 30 % or below. Likewise, in the case of the e-coli strains, lowering an antibiotic producer’s attack rate, the number of sensitive strains it kills per contact actually increases the producer’s numbers. Dropping down the producer’s attack rate below 20 % dramatically increases the numbers of producers. Much as we saw in the simulation (Fig. 8), researchers found empirical evidence that producers of antibiotic that restrain their attack (a mutation of producers that has a lower attack rate) actually increase in abundance, a phenomenon in cyclic dominance known as “survival of the weakest” [61, 79]. In both the public health game and the microbial game, this “survival of the weakest” effect results from a multiple types of negative feedback captured by the phrase, “the enemy of my enemy is my friend” an ancient adage still in use in political rhetoric because it seems to describe actual patterns of strategic conflict [80].

The antibiotic producer’s enemy is the resistant strain. The enemy of the resistant strain is the susceptible strain (“grower”) which is normally attacked by the producer of the antibiotic. So if the producer instead restrains its attack to a very low level, then the susceptible strain grows very quickly and wipes out the resistant strain. Now the producer’s growth is unrestrained. Likewise in the public health game, the enemy of infection is health protection, because protection curbs local infection in the short run. However, an enemy of protection is healthy defectors, because healthy defectors can do very well, in the short run, thus “persuading” protectors to defect. Then defectors become the victim of their own success, much as we saw in the very simple example in Fig. 1, in which the more successful defector persuaded others to defect. Lowering infectivity can help defectors do well by making it possible to defect and yet be healthy. As a result, defection rises, increasing vulnerability to infection. Thus reducing risk of infection by reducing infectivity can lead to a compensation, if individuals compensate for lower infectivity by switching from protection to defection.

A somewhat similar effect seems to operating in “risk compensation,” in which individuals who perceive lower risk in turn lower their protective behavior [81]. Individuals may learn that using anti-retroviral therapy (ART) lowers infectivity [10, 82–84]. However, believing that infectivity is lowered by the ART apparently leads many to decrease health protective behavior such as using a condom or limiting the number of one’s partners [10, 82, 83, 85, 86]. One component of this feedback loop is risk compensation, an increase in unsafe behaviors in response to perceptions of decreased risk caused by introduction of preventive or treatment interventions (“harm reduction” interventions) [81], e.g. seatbelt wearing increasing reckless driving [87], use of sunscreen increasing sun exposure [88], as well as ART increasing unprotected sex. These cases of risk compensation are a type of policy resistance that we can better understand with systems modeling that include feedback [4–6, 89–91]. Fearing that harm reduction can lead to increase risk taking, healthcare providers, policy makers or citizens may oppose such approaches. For example, Healthcare providers sometimes resist wide distribution of drugs such as Naloxone that reduce drug overdose deaths because it might be perceived as a “safety net” encouraging for risker drug use [92]. Although my model did not attempt to model this variety of health behaviors, the results suggest the need to create dynamic models for a variety of these other behaviors to see if similar results apply. In any case, it seems that the rational policy response is to provide incentives to increase protective behavior in addition to any treatments that reduce harm. However, some worry that preventive or curative treatments to infection will undermine behavioral restraint that normally result from fear of a rise in infection [83]. The most pernicious form of this belief is that infection is punishment for what they see as behavioral transgression [93, 94]. From such an intolerant standpoint, the negative feedback loop between disease and abstinent behavior is desirable, and a cure would negate that loop. Thus, research that identifies individuals’ perceptions of feedback loops, including perceived benefits, costs and risks, may uncover morally charged values. Making explicit assumptions, values and perceived causal feedback loops, through a combination of qualitative methods and system modeling [95] seems an effective approach for engaging groups of stakeholders and establishing common understandings in economic and ecological development [96, 97], and may prove effective in advancing a participative-collaborative understanding of resilience in social-ecological systems [98]. Such a discussion begins by making assumptions explicit.

The assumptions of the models I presented are highly simplified in that we assume everyone follows the same simple rules given the situation they face. This simplification allowed us to conclude that complex patterns of resilience, cyclic dominance and hydra effects can emerge even with these simple assumptions, e.g. we do not need very complex rules and individual differences to yield complex results. When individuals take action, this affects the situation of others, thus we could thoroughly explore a dynamic missed in more static behavioral models. However, the simplicity of the dynamic rules in the models I presented is also a limitation, and future work should address variants of the models. We systematically explored how results change when we vary perceived benefit, vary infectivity and attack rate, and vary network structure. To some extent, we can infer that the results for low infectivity or zero infectivity resemble what happens when individuals can become immune. We saw that hydra effects occur for infectivity near zero, possibly suggesting that hydra effects may occur when individuals acquire immunity as well. However, we did not explore vaccination behavior. Future work should systematically examine what happens when individuals acquire immunity through vaccination, e.g. do we still see the types of resilience and resistance? As a starting point, this paper has demonstrated some ways that resilience and resistance are forms of balance that can be tipped by disrupting local community feedback, either by breaking up local links directly or by altering the benefits, costs and risks. This modeling perspective, along with participative discussion, may lead to more effective, if counter-intuitive approaches to interventions, e.g. ways of tipping the status quo equilibrium, or countering hydra effects. Methodologically, agent-modeling combined with longitudinal data and, if possible, social network data on perceived costs, benefits and behaviors, should help researchers should researchers spot the types of non-linear effects identified in this paper. Standard assumptions of linear effects of interventions can interfere with finding non-linear effects, such as tipping points, policy resistance and hydra effects.

## Additional file

Additional file 1: Figure S1. Lowering the costs for producing antibiotic has the effect of increasing resistance. At first this effect seems surprising, but what is happening is that by giving a cost advantage to the producer, the grower, the enemy of the producer decreases. Also the producer’s predator, the resister, increases in abundance because its own predator has decreased. **Figure S2.** When producer’s (purple) attack rate is set at a very low level (10 % here), the producer allows its prey (grower, shown in green) to multiply, leading to extinction of resister (red), allowing the producer to take over. In this example of run with the producer attack rate set at 10 %, the producer takes over by 28 steps. (DOCX 106 kb)
