# Supplementary material for: Resilience, tipping, and hydra effects in public health: emergent collective behavior in two agent-based models
Source: BMC Public Health. 2016 Mar 15;16:265. doi: 10.1186/s12889-016-2938-8 (PMC4791925; doi:10.1186/s12889-016-2938-8)
Supplement: Additional file 1: Figure S1. — Lowering the costs for producing antibiotic has the effect of increasing resistance. At first this effect seems surprising, but what is happening is that by giving a cost advantage to the producer, the grower, the enemy of the producer decreases. Also the producer’s predator, the resister, increases in abundance because its own predator has decreased. Figure S2. When producer’s (purple) attack rate is set at a very low level (10 % here), the producer allows its prey (grower, shown in green) to multiply, leading to extinction of resister (red), allowing the producer to take over. In this example of run with the producer attack rate set at 10 %, the producer takes over by 28 steps. (DOCX 106 kb) [file 12889_2016_2938_MOESM1_ESM.docx]

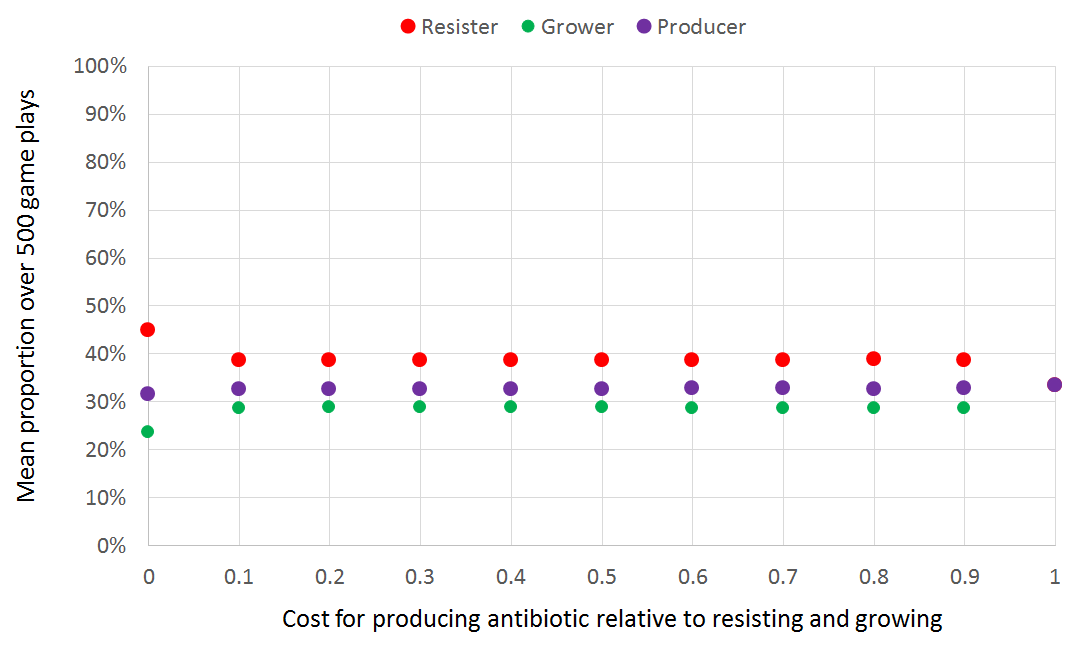


Figure S1. Lowering the costs for producing antibiotic has the effect of increasing resistance. At first this effect seems surprising, but what is happening is that by giving a cost advantage to the producer, the grower, the enemy of the producer decreases. Also the producer’s predator, the resister, increases in abundance because its own predator has decreased.


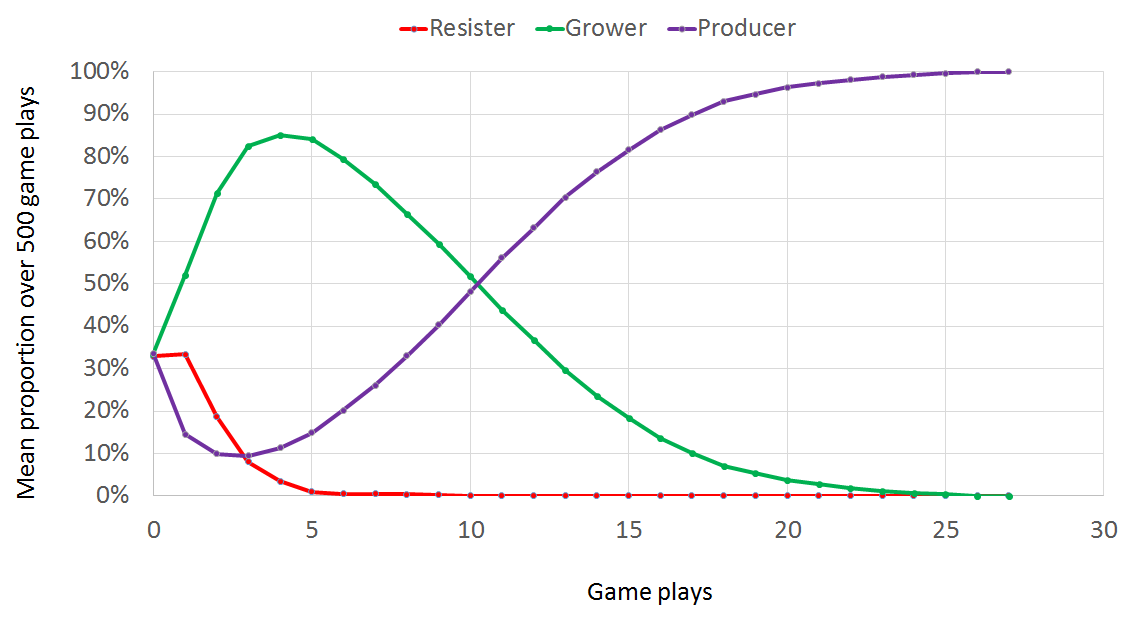


Figure S2. When producer’s (purple) attack rate is set at a very low level (10% here), the producer allows its prey (grower, shown in green) to multiply, leading to extinction of resister (red), allowing the producer to take over. In this example of run with the producer attack rate set at 10%, the producer takes over by 28 steps.
